# A Resource-Rational Account of Human Eye Movements During Immersive Visual Search

**DOI:** 10.1162/OPMI.a.322

**Published:** 2026-02-01

**Authors:** Angela Radulescu, Bas van Opheusden, Frederick Callaway, Thomas L. Griffiths, James M. Hillis

**Affiliations:** Center for Computational Psychiatry, Icahn School of Medicine at Mount Sinai, New York, NY, USA; OpenAI; Department of Psychology, New York University, New York, NY, USA; Departments of Psychology and Computer Science, Princeton University, Princeton, NJ, USA; Meta

**Keywords:** visual search, virtual reality, resource rationality, deep reinforcement learning

## Abstract

The nature of eye movements during visual search has been widely studied in cognitive science. Virtual reality (VR) paradigms are an opportunity to test whether computational models of search can predict naturalistic search behavior. However, existing ideal observer models are constrained by strong assumptions about the structure of the world, rendering them impractical for modeling the complexity of environments which can be studied in VR. To address these limitations, we modeled immersive visual search as a reinforcement learning problem, in which sequential decisions are made over a multidimensional representation of the environment learned by a convolutional neural network. In our formulation, RL agents learned a policy over latent states—effectively solving what is known as a meta–Markov decision process (meta-MDP), where each decision concerns how to allocate attention to information in the environment. Training deep-RL agents on the meta-MDP showed that learned (i.e., optimal) search policies converge to a classic ideal-observer model of search developed for simple (1D) stimuli. We compared the learned resource-rational policy with human gaze data from a visual-search experiment conducted in VR and found qualitative and quantitative alignment between model predictions and human behavior. However, both the model’s simulated performance and its correspondence with human behavior depended strongly on the representational features available to the policy. These results suggest that naturalistic visual search behavior can partially be explained by resource-rational allocation of limited cognitive resources, and the choice of representation influences the degree of alignment between model and human behavior.

## INTRODUCTION

Developing models of human visual search is a long-standing problem in cognitive science (Chun & Wolfe, 2005; Eckstein, 2011; Yarbus, 2013). Visual search is a common task in which humans look for an item (“target”) in a crowded visual environment that consists of various “distractors.” While visual search tasks differ in how targets and distractors are defined, they all require a searcher to sample information from the environment. Such sampling may occur overtly, by moving one’s eyes to different parts of the environment; or covertly, by fixating at a location and deploying attention to sample (Engelke et al., 2014). For instance, to find our keys in a cluttered room, we may have to move our eyes to different regions of the scene, and fixate to process local details. Generally, visual search requires selectively sampling information from the environment, under time and resource constraints, in service of a goal.

Visual search has been studied in behavioral paradigms that approximate real-world search using static 2D displays and highly restricted head movement. This research has identified different features that drive eye movements (Henderson, 2020; Treisman & Gelade, 1980; Wolfe & Horowitz, 2017), and has led to the development of several theoretic accounts of search (Acharya et al., 2017; Butko & Movellan, 2008; Hayhoe & Ballard, 2005; Hoppe & Rothkopf, 2019; Najemnik & Geisler, 2005; Sprague & Ballard, 2003). One important insight from this work is that “ideal observer” models, which assume that eye movements are optimally selected to gather statistical information, predict behavioral and neural signatures of information processing in multi-sensory perception tasks (Alais & Burr, 2019; Ernst & Banks, 2002; Hillis et al., 2004; Landy et al., 2012; Sebastian et al., 2017, 2020; Walshe & Geisler, 2022). However, because these ideal observer models have been tailored to specific paradigms with well-defined environment structure, they are typically constrained by statistical assumptions about the visual input (Najemnik & Geisler, 2005).

Recent work has begun to leverage virtual reality (VR) to study visual search in naturalistic settings (Beitner et al., 2021; Bennett et al., 2021; Li et al., 2016). This opens up the question of whether ideal observer models can generalize to environments in which humans have to search for suprathreshold, 3D targets in an active-viewing, embodied setting. VR affords improved ecological validity, enhancing perception above and beyond 2D environments, and enabling guidance by naturalistic spatial priors (Beitner et al., 2021; Schubring et al., 2020; Tian et al., 2021). But instantiating ideal observer models of visual search in VR is challenging, because such models do not specify what the relevant features for search should be in the real world, nor do they provide a clear account of what objective the agent should optimize when searching through the space of those features.

In this work, we address these challenges by framing eye movements during immersive visual search as a trade-off between gathering information and the cost of the associated computation (Gershman et al., 2015; Griffiths et al., 2015; Howes et al., 2009; Lieder & Griffiths, 2020). This approach—analyzing cognition as the rational allocation of limited resources, or “resource-rational analysis”—has been used to generate models of a range of cognitive processes, explaining observations from research on judgment (Lieder, Griffiths, & Hsu, 2018; Lieder, Griffiths, Huys, et al., 2018), decision-making (Callaway et al., 2021; Gul et al., 2018), and planning (Callaway et al., 2022). We leverage resource-rational analysis in combination with convolutional neural networks (CNNs) and deep reinforcement learning to specify and train agents that search for objects in a naturalistic, high-dimensional visual search task. We compare the behavior of the trained agents to human gaze data in a visual search experiment conducted in VR.

By training agents to perform visual search over a representation of the environment encoded by a deep neural network, we demonstrate that previously proposed ideal observer models are optimal under more general assumptions that include a formal specification of the structure of naturalistic scenes. We also show evidence that the resource-rational policy aligns with human gaze behavior in an active-viewing, immersive 3D setting. And we use this framework to systematically ablate features of the model in order to study which feature spaces guide search in naturalistic contexts. Our findings suggest that naturalistic visual search involves sequential information gathering decisions that are resource-rational, and operate over a structured representation of the environment.

## RESULTS

### Experiment: Immersive Visual Search

**Figure 1.**
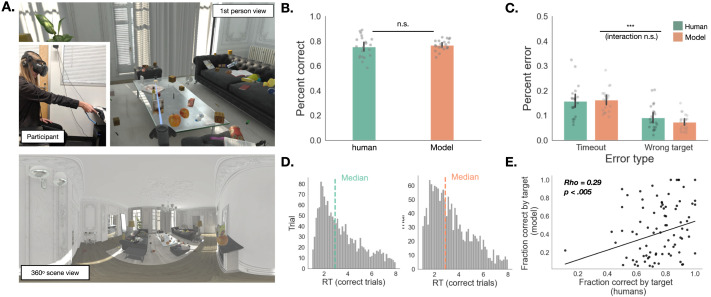
**Immersive visual search task**. **A: Top:** Egocentric view of the participant shown in the inset during the immersive visual search task. The participant is using the controller to report having found the target (the white pizza box). Participants experienced the scene from a fixed viewpoint, and were free to move their eyes and head to sample different parts of the environment. **Bottom**: 360-degree equirectangular projection of the scene above. **B:** Comparison of percent correct across all trials of the experiment (human participants vs. computational model). **C:** Comparison of percent error, as a function of whether a wrong target was reported, or the trial timed out (human participants vs. computational model). **D:** Empirical reaction time distribution (left) compared to the reaction time distribution simulated using the computational model (orange). **E:** Correlation between actual and simulated average fraction correct as a function of target.

To address the question of whether ideal observer models of search generalize to naturalistic environments, we conducted a study of visual search in virtual reality (Figure 1A, Immersive Visual Search Task). Human participants were immersed in virtual indoor scenes and interacted with the environment using a handheld controller while their gaze was tracked. When immersed in a scene, the participant’s goal was to find a target object among several distractors scattered around the room. Each scene was uniquely defined by the room context (e.g., living room), viewpoint (e.g., above the table), target (e.g., pizza box) and distractor set.

Despite the complexity of the environment and the relatively large number of distractors, participants successfully found the target object 75% of the time, fixating an average of 12.6 items before locating the target (Figure 1B). Of the error trials, 9% were due to participants reporting the wrong target, while 16% were due to participants running out of time to search (Figure 1C). More than half of all successful searches were under 3 seconds long, suggesting that people are able to efficiently search for objects in complex 3D environments (Figure 1D). We next asked what strategy they use to accomplish this. In particular, is their fixation behavior consistent with an ideal observer model? To address the question of how people select which object to fixate next, we must first specify how objects are represented.

### Defining a Feature Space for Search

In traditional visual search tasks, simple 2D targets are typically presented overlaid on unstructured noisy backgrounds. By contrast, in naturalistic visual search, targets are complex 3D objects that can appear in multiple orientations, scales, and lighting conditions. This necessitates searching for objects that match the target on a set of abstract features that are invariant across pose and illumination—that is, a representation. From a modeling perspective, the choice of representation encodes a *hypothesis about what prior knowledge humans bring to the task*. To specify this knowledge, we compared two approaches: an explicit approach based on shape and color, and an implicit approach using neural networks.

**Figure 2.**
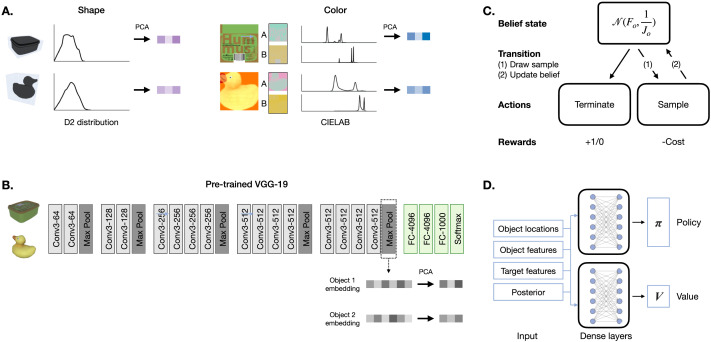
**Modeling approach**. The feature space for visual search consists of objects and their perceptual features. Perceptual features were extracted in two different ways (Feature Extraction section): explicitly, by summarizing each object’s shape and color information; and implicitly, by embedding objects in a feature space defined by activation in the layers of a pre-trained convolutional neural network (CNN). **A:** Left, mesh representation and corresponding shape distributions. Right, 2D texture and corresponding CIE L*a*b* color distributions. **B:** Architecture of VGG-19, the pre-trained convolutional neural network used to extract object embeddings. **C:** Graphical representation of the meta-MDP model of visual search. *F*_*o*_ represents a vector of mean beliefs for a particular object and 1/*J*_*o*_ represents a vector of precisions around those beliefs. **D:** Architecture of the deep reinforcement learning agent trained to solve the meta-MDP. A policy and value network each received a set of inputs consisting of object locations, object features, target features and the posterior over which object is the target.

Previous work in visual cognition has shown that during visual search, eye movements are guided by top-down attention to perceptual features of the target object (Wolfe & Horowitz, 2017). But which perceptual features do people use? Two likely candidates are shape, which we quantified using the D2 distribution, a measure of global geometric properties for 3D objects (see Feature Extraction section); and color, which we quantified in the CIE L*a*b* space (see Feature Extraction section). Each of these representations encodes each object as a point in a high-dimensional perceptual space (Figure 2A).

**Figure 3.**
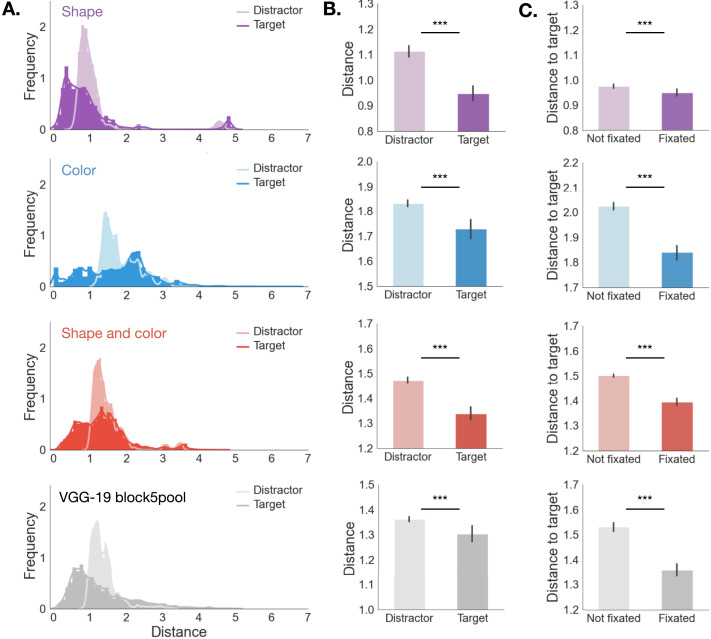
**Distance to target in feature space is predictive of gaze**. **A:** Distribution of distances between the features of currently fixated object and the features of either the target (darker shade) or the average features of all distractors (lighter shade). This distribution was computed separately for the shape, color, shape + color and VGG-19 feature spaces. **B:** Means and 95% CIs of the distributions in **A**. **C:** Distance to target for all objects in a scene as a function of whether the object was fixated or not. For the shape, color and shape + color spaces, distance was computed as Euclidean distance. For the VGG-19 embedding, distance was computed pairwise between object as $\frac{\left(1-\rho \right)}{2}$, where *ρ* is the Spearman correlation coefficient. ***: *p* < .0001.

As an initial validation of these two feature spaces, we asked whether the representation can predict fixations in a model-agnostic way. First, we asked whether every time that a participant looks at an object, the object being fixated is more similar in feature space to the target than to the average of all distractors in the scene (Figure 3A and B). A paired-samples *t*-test was conducted to compare distance between the fixated object to target and to the average of all distractors, respectively. For shape, we found a significant difference between the distance to target (*M* = 0.95, *SD* = 0.07) and to distractor average (*M* = 1.11, *SD* = 0.05); *t*(20) = −17.12, *p* < .0001, *d* = 2.63. Similarly for color, we found a significant difference between the distance to target (*M* = 1.73, *SD* = 0.09) and to distractor average (*M* = 1.83, *SD* = 0.03); *t*(20) = −5.52, *p* < .0001, *d* = 1.49. Finally, for shape and color, we found a significant difference between the distance to target (*M* = 1.33, *SD* = 0.69) and to distractor average (*M* = 1.47, *SD* = 0.47); *t*(20) = −12.83, *p* < .0001, *d* = 2.71.

We further reasoned that if participants used the features we identified in order to search, the distance between the objects present in a scene and the target should be modulated by whether objects were fixated or not (Figure 3C). A paired-samples *t*-test was conducted to compare distance to target between fixated and non-fixated objects. For shape, we found a significant difference in distance to target between fixated (*M* = 0.95, *SD* = 0.03) and non-fixated objects (*M* = 0.97, *SD* = 0.02); *t*(20) = −3.69, *p* < .0001, *d* = 0.95. Similarly for color, we found a significant difference in distance between fixated (*M* = 1.84, *SD* = 0.06) and non-fixated objects (*M* = 2.02, *SD* = 0.04); *t*(20) = −13.37, *p* < .0001, *d* = 3.39. Finally, for shape and color, we found a significant difference in distance between fixated (*M* = 1.4, *SD* = 0.03) and non-fixated objects (*M* = 1.5, *SD* = 0.01); *t*(20) = −14.16, *p* < .0001, *d* = 4.01. In other words, objects that were fixated during search were more likely to be similar to the target than objects that were not fixated. This was the case for both shape and color, as well as the conjunction of shape and color. Taken together, these results suggest that the explicit feature space captures some of the underlying perceptual representations that people use to guide search.

Although the previous analysis shows that people are sensitive to both shape and color when choosing where to fixate, people could be drawing on a richer representation of the objects. To address this possibility, we also considered a third feature space that was learned by a convolutional neural network (CNN). Specifically, we passed a 2D image of each object through the perceptual module of a pre-trained VGG-19 network (Simonyan & Zisserman, 2014) trained on image classification (Figure 2B, Feature Extraction section). CNNs such as VGG-19 have been proposed as models of the human visual system (Battleday et al., 2020; Lindsay, 2021). Based on previous literature, we computed object embeddings from the second-to-last classification layer of VGG-19 (Fan et al., 2020; Long et al., 2024). However, we reasoned that because these features are optimized for classification, features in the last perceptual layer might be better suited for visual search. We tested this directly, and found that even though distance computed from both VGG-19 embeddings was predictive of gaze, the embedding computed from the perceptual layer was overall a better predictor of gaze than the embedding computed from the classification layer (Supplementary Information Section 5.1). As such, we used the perceptual embedding in subsequent analyses.

Our findings for the VGG-19 embedding mirrored our previous results (Figure 3): we found a significant difference between the distance to target (*M* = 1.3, *SD* = 0.076) and to distractor average (*M* = 1.36, *SD* = 0.02); *t*(20) = −3.42, *p* < .005, *d* = 1.01. We also found a significant difference in distance between fixated (*M* = 1.36, *SD* = 0.05) and non-fixated objects (*M* = 1.53, *SD* = 0.04); *t*(20) = −14.66, *p* < .0001, *d* = 3.47.

Finally, a challenge for agents learning in multidimensional environments is identifying a low-dimensional state representation that supports behavior (Leong et al., 2017; Niv, 2019; Radulescu et al., 2019). To compute this representation for our setting, we applied principal component analysis (PCA) to each candidate feature space (shape, color, and VGG-19 embedding). We verified that the similarity structure of objects was preserved for all three feature spaces, even when retaining a relatively small number of principal components (Supplementary Information Section 5.2). Given that fixated and non-fixated objects were discriminable in all three feature spaces (Figure 3), we considered all three of these as possible representations in the following analyses.

In addition to validating our choice of representation, these results indicate that people are using a strategy that fixates objects that are similar to the target. This is consistent with the classic ideal observer models of visual search. However, to demonstrate this rigorously, we must determine what exactly constitutes an ideal observer in our task.

### Defining the Search Problem

Representing target and distractor objects in abstract feature spaces is likely necessary to conduct a visual search in a 3D environment. But it also poses a problem for traditional ideal observer models, which assume that search targets have unstructured image statistics—that is, they can be parameterized as Gaussian and IID (Najemnik & Geisler, 2005). Because we are studying the more general case in which these assumptions do not hold (Supplementary Information Section 5.3), it is unclear what constitutes “ideal” observing during immersive visual search. To answer this question, we first formally define the problem that the searcher is solving.

The key intuition behind our analysis is that visual search can be cast as a sequential decision problem (Acharya et al., 2017; Butko & Movellan, 2008; Hayhoe & Ballard, 2005; Hoppe & Rothkopf, 2019; Najemnik & Geisler, 2005; Sprague & Ballard, 2003). In this view, search consists of a sequence of decisions about where to fixate next, and each such decision takes into account the information gained from previous fixations. To capture the tradeoff between the utility and cost of information gathering, we formalize visual search as a *metalevel Markov decision process* (Hay et al., 2012; Russell & Wefald, 1991). Like a standard MDP (Sutton & Barto, 2018), a meta-MDP consists of a set of states, a set of actions, a transition function, and a reward function. Unlike in a standard MDP, however, the states correspond to beliefs and the actions correspond to computations. The transition function describes how computations update beliefs. Finally, the reward function incentivizes decisions made based on accurate beliefs, but also penalizes computation. Below, we describe a meta-MDP for a general version of visual search (Figure 2C, see Full Meta-MDP Specification section for the full model specification).

- **Latent state**: A visual scene is defined as a set of objects at specific locations, with each object represented as a feature vector in a low-dimensional space. We assume that at the beginning of each search episode, the agent can perceive all objects and knows their exact location, but does not have access to the features of the objects.
- **Belief states**: A belief is a distribution over latent states. For every object, the agent’s current belief is given by two vectors, representing the current mean and precision of that object’s features.
- **Computations**: A computation corresponds to fixating a particular object and sampling information about its visual features. A special computation indicates that search should be terminated (see Reward).
- **Transition function**: The transition function specifies what information is sampled by each computation (the features of objects near the center of gaze) and also how that information is incorporated into beliefs (Bayesian cue combination). Each sample incorporates a fovea-like filter, such that attention paid to each object in the scene exponentially decreases with its distance from the object currently being fixated.
- **Reward**: The agent incurs a cost for each computation. After a computation, it calculates a posterior probability over which object is the target given the current belief state. Search terminates when the maximum of the posterior exceeds a threshold. Upon termination, the agent reports the object most likely to be the target and receives a reward if this is correct and no reward otherwise.

The meta-MDP assumes the state of the environment does not change, but the agent’s beliefs about the state of the environment do. The negative reward implements the “cost” of gathering information about the state, and incentivizes solutions that balance utility (in this case, fixating objects similar to the target) and complexity (making only as few fixations as necessary). A meta-MDP shares similarities with a partially observable Markov decision process (POMDP), a point which we return to in the Discussion.

### Learning a Resource-Rational Search Policy

By expressing visual search as a meta-MDP over a complex representation of the environment, we can ask, what is the resource-rational (optimal) policy that maximizes performance while minimizing the cost of search? To address this question, we trained artificial agents to solve the meta-MDP using deep reinforcement learning. We provided artificial agents with the states, computational actions, rewards/costs and transition function that define the dynamics of the environments experienced by human observers, and used proximal policy optimization (PPO) (Schulman et al., 2017) to discover an optimal policy (Figure 2D, Solving the Meta-MDP section). Deep reinforcement learning allows us to move beyond scenarios where the optimal policy is analytically computable based on simplifying assumptions. As is standard practice in deep reinforcement learning, we initially fixed the representation to VGG-19 with 6 principal components. This choice was made for pragmatic reasons (convergence and training speed), based on tuning runs that yielded high task performance and stable convergence.

**Figure 4.**
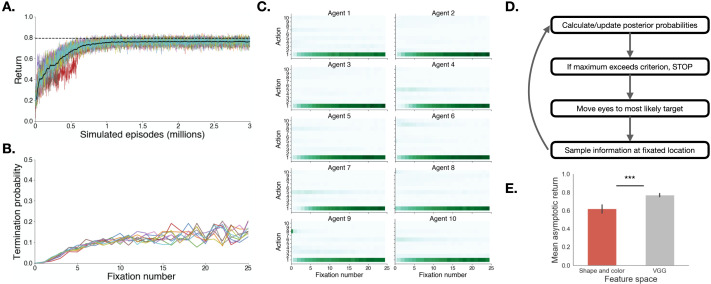
**Deep RL training results**. **A:** Learning curves for 10 agents trained to solve the meta-MDP using deep reinforcement learning over the VGG-19 feature space. Note that all agents converge after about 1 million episodes. The dotted line represents the average return obtained by the *Fixate_MAP* policy depicted in **D**, over 10,000 simulations with an optimal stopping criterion. **B:** Probability of terminating search as a function of the number of fixations previously taken (i.e., timepoint within an episode), for the 10 different networks. **C:** Frequency of chosen action, as a function of timepoint (*x*-axis) and rank in posterior probability space (*y*-axis). Action 1 corresponds to fixating on the object most likely to be the target, action 2 to the object second most likely target, and so on. Each panel represents one of the 10 trained agents. All 10 agents converge to *Fixate_MAP* (choosing the object most likely to be the target) within the first 5 fixations. **D:** Graphical depiction of the *Fixate_MAP* policy that agents learned. **E:** Average asymptotic return as a function of feature space (shape and color vs. VGG-19).

We trained 10 independent artificial agents and inspected their returns and policy (Figure 4). We found that all 10 agents achieve asymptotic high returns after about a million training episodes (Figure 4A). Moreover, agents converged to a policy in which they terminated search whenever the maximum posterior belief exceeded a threshold (Figure 4B) and consistently fixated the object most likely to be the target (Action 1 in Figure 4C). We term this policy *Fixate_MAP*, shorthand for fixating the *maximum a posteriori* (MAP) target given the agent’s current belief state. All agents converged to similar returns, which were approximately equal to that of a hard-coded *Fixate_MAP* policy (dotted line in Figure 4A).

We repeated this training procedure with 10 additional agents, this time using the explicit representation defined by shape and color features, reduced to 6 principal components. While returns were lower on average then for the VGG-19 representation, we replicated the finding that agents converged to a policy that approximates *Fixate_MAP* (Supplementary Information Section 5.4). Interestingly, asymptotic returns were significantly higher for agents that learned over the VGG-19 representation (Figure 4E, *t*(12) = −5.74, *p* < .0001, *d* = 2.57, 95% *CI* = [−0.21, −0.09]). This effect size translates to a difference of 0.15: in other words, the 10 agents trained on the VGG-19 representation achieved returns that were 0.15 larger on average than the 10 agents trained on the shape and color representation.

Notably, the *Fixate_MAP* policy that the agents learned incorporates the previously proposed “ideal observer” model for visual search in simple environments as a special case. For these simple environments, the ideal detector is an inner product of the image with a fixed template (Najemnik & Geisler, 2005), which we can interpret in our meta-MDP as a 1D feature space. By using deep reinforcement learning to train agents to solve the more general version of visual search, we provide a rational basis for this policy in structured, naturalistic settings. Despite differences in representational assumptions, all agents converged to the same *Fixate_MAP* policy, suggesting that *Fixate_MAP* is close to the optimal policy for the meta-MDP. We therefore use *Fixate_MAP* as a computationally tractable approximation of the optimal policy in the analyses that follow.

### Effects of Representation on Agent Performance

While all deep reinforcement learning agents converged to *Fixate_MAP*, our previous analysis suggested that representational choices (i.e., which feature space is used to train the RL agent) significantly influence task performance. In other words, *Fixate_MAP* is optimal within the representational constraints it is given.

This raises the question of how sensitive *Fixate_MAP*’s performance is to the choice of representation. To systematically answer this question, we conducted an “ablation study”: we simulated behavior from agents that behave according to *Fixate_MAP* over different feature spaces, varying the number of principal components, and optimizing the termination threshold for each resulting representation (Ablation Study section, Threshold Optimization section). To test for differences in model performance, we used a mixed-effects approach. We first simulated *Fixate_MAP* with an optimized threshold for each representation, quantified as feature space (shape, color, shape and color and VGG-19) and number of principal components used in simulation (1–7). We then fitted a linear mixed effects model with average return and number of fixations across scenes as dependent variables. Independent variables were feature space and number of principal components. We also included random intercepts for “subject” (where “subjects” are simulated agents); and an interaction term between feature space and number of principal components.

We note that because this is a simulation study, variance in our metrics could, in the limit, be reduced to near 0. As such, the results of our statistical analysis can only be interpreted in the context of this simulation. However, because we were interested in what the effect sizes would be for an experiment with a sample size comparable to the one we set out to model, we simulated each unique agent 20 times. This would correspond to 20 “agents” that behave according to *Fixate_MAP* for all scenes in our experiment, given a unique combination of feature space and number of principal components.

**Figure 5.**
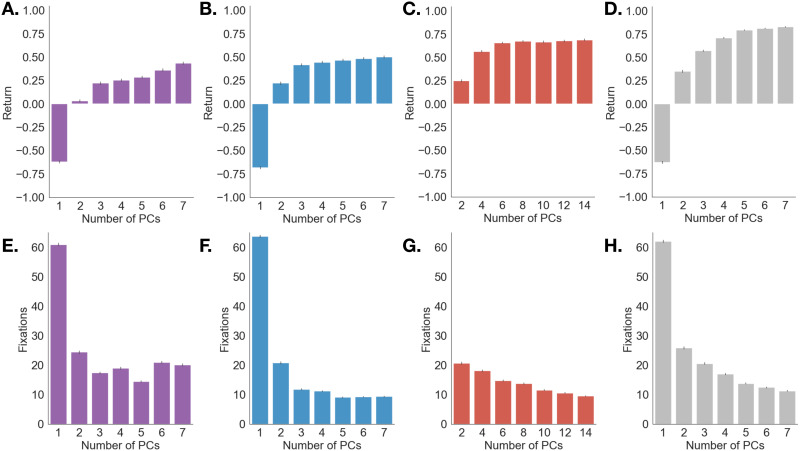
**Agent performance as a function of belief state representation and number of principal components**. Performance metrics were computed over 20 simulations of the *Fixate_MAP* policy per combination of feature space (purple: shape; blue: color: red: shape and color; grey: VGG-19) and number of principal components. **A–D:** Average return. **E–H:** Average number of fixations.

This analysis revealed that the choice of representation significantly impacted agents’ search performance, quantified both as return and number of fixations taken before reaching the goal (Figure 5).

#### Agent Returns Are Sensitive to Belief State Representation.

An omnibus Type III test revealed a main effect of feature space: feature space significantly predicted average return, (*χ*^2^(3) = 2639, *p* < .0001). Including number of principal components as a predictor also improved model fit, (*χ*^2^(6) = 44085, *p* < .0001), suggesting that the number of principal components used in the belief state impacts model performance. Interestingly, adding the feature space × number of principal components interaction also significantly improved model fit (*χ*^2^(18) = 12004, *p* < .05), suggesting that differences in return across feature spaces depended on how many principal components were used. Given this interaction, and since visual inspection suggested diminishing marginal returns with the number of principal components for 3 out of 4 feature spaces (Figure 5A–D), we performed post hoc comparisons of estimated marginal means to determine at which point including information from additional principal components did not improve returns.

**Figure 6.**
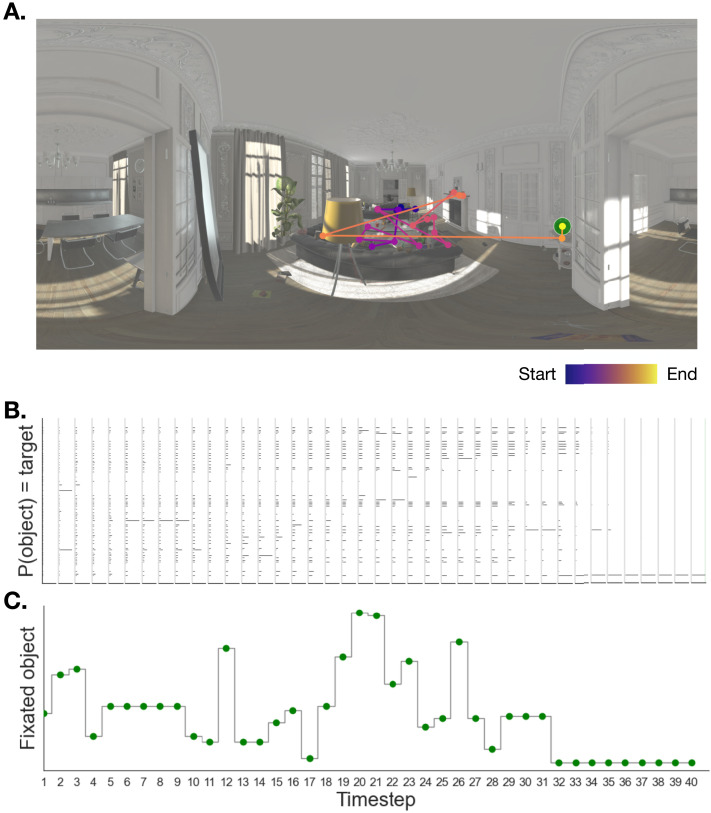
**Example of agent behavior**. **A:** Sequence of fixations obtained by simulating agent behavior from the *Fixate_MAP* policy once in one of the living room scenes. **B:** Evolution of the posterior distribution over target object at each timestep for the agent shown in **A**. Height of each bar represents the posterior probability of that particular object being the target object. **C:** Fixated object at each timestep (note the agent always fixates the object most likely to be the target).

We found that the best-performing model used the VGG-19 representation and 7 principal components, and achieved significantly higher returns than a model that used the shape and color representation and the equivalent number of principal components, 7 each for shape and color (*b* = 0.14, *SE* = 0.0087, *z* = 16.32, *p* < .0001, compare the highest bars in Figures 5C and 5D). This is consistent with our previous analysis of asymptotic returns (Figure 4E). However, this same model did not perform significantly better than a VGG-19 model that used one fewer principal components (*b* = 0.018, *SE* = 0.0087, *z* = 2.11, *p* = 0.95, compare the last two bars in Figure 5D), suggesting diminishing returns in performance with more principal components. In other words, of the feature spaces we tested, the *Fixate_MAP* policy achieved maximal returns over a 6-dimensional VGG-19 representation. A sample fixation trajectory simulated from this policy is shown in Figure 6.

Finally, even though shape on its own achieved the lowest returns, combining shape and color features (7 PCs each) gave agents a marginal advantage above and beyond only using color (7 PCs) (*b* = −0.19, *SE* = 0.0087, *z* = −22.02, *p* < .0001, compare the highest bars in Figures 5B and 5C). This finding is not surprising given that, for most objects, the retinal projection is a highly ambiguous signal that maps to 3D shape. Therefore, unlike in research using a fixed set of 2D spatial patterns, shape on its own may not be a useful feature for many visual search tasks characterized by natural 3D scene perception. However, when used in conjuction with color in our experiment, shape statistics provide a useful discriminative space for search.

#### Number of Fixations Depends on Belief State Representation.

To examine how the choice of representation impacted search time, we repeated our mixed-effects analysis using number of fixations as an outcome variable. We found that feature space significantly predicted number of fixations, (*χ*^2^(3) = 2820, *p* < .0001). Including number of principal components as a predictor also improved model fit, (*χ*^2^(6) = 69378, *p* < .0001), suggesting that the number of principal components used in the belief state impacts the amount of fixations that agents spent searching. Again, adding the feature space × number of principal components interaction also significantly improved model fit (*χ*^2^(18) = 39449, *p* < .0001), suggesting that differences in number of fixations across feature spaces depended on how many principal components were used.

In post hoc comparisons, we found that the models that spent the fewest fixations searching were the ones that used either the color feature space with 7 PCs or the shape and color representations with 7 PCs each (*b* = −0.18, *SE* = 0.23, *z* = −0.79, *p* = 1, compare the lowest bars in Figures 5F and 5G), followed by the VGG-19 model that used 7 PCs (*b* = 1.73, *SE* = 0.23, *z* = 7.39, *p* < .0001, compare the lowest bars in Figures 5G and 5H). So while models that used the explicit shape and color representation achieved lower returns, they also used about 2 fewer fixations on average. This sensitivity of agents’ behavior to choice of representation suggests that in naturalistic environments, the speed-accuracy trade-offs that are characteristic of visual search can at least partly be attributed to the feature space that agents use to search (Heitz & Schall, 2012).

### Predicting Human Behavior

Our analysis of the policies learned by the meta-MDP suggests that *Fixate_MAP* is a near-optimal solution to the naturalistic visual search task defined here. The ablation study also demonstrates an effect of belief state representation on the performance of the *Fixate_MAP* policy. But is the learned *Fixate_MAP* policy consistent with human behavior? And if so, which representation best explains human gaze patterns? To answer these questions, we again took an ablation approach, this time simulating behavior from the policy in order to compare it to human behavior in the immersive visual search experiment.

#### Model Qualitatively Captures Human Accuracy, Errors, and Reaction Times.

We first compared the accuracy (defined as fraction correct), error patterns (timeouts and false alarms), and reaction time distributions obtained in simulation to those observed in the experiment. We simulated the full experiment with a fixed setting of the parameters (Full Meta-MDP Specification section), assuming a linear relationship between number of fixations and reaction time (Comparing Human and Agent Policies section).

We found that model performance quantified as percent correct is statistically indistinguishable from human performance (Figure 1B, *t*(80.8) = .53, *p* = 0.60, 95% CI [−0.02, 0.035]). To test whether error patterns were similar across models and humans, we fit a linear mixed effects model that predicted average percent error, and included error type (timeout vs. wrong target), agent type (human vs. model), and random intercepts for each participant (Figure 1C). We found that the interaction was not significant (*χ*^2^(1) = 1.05, *p* = 0.31), suggesting that sources of error did not significantly differ between model and humans. We found a main effect of error type (*χ*^2^(1) = 18.0, *p* < .0001), with timeouts being more frequent than reporting the wrong target (*t*-ratio(40) = −7.03, *SE* = 0.011, *p* < .0001). Finally, the model was able to reproduce the shape of the RT distribution we observed in the human data (Figure 1D), with the median reaction time on correct trials slightly higher in humans (2.95, *IQR* = 1.98–4.51) than in agents (2.88, *IQR* = 1.92–4.38). Notably, supplementary analyses indicated similar overall accuracy for humans and the model, but performance varied across contexts in different ways (Supplementary Information Section 5.6).

To gain insight into whether and how the model might diverge from human behavior, we also tested whether the model is more likely to be correct on the same target that humans are. We found a statistically significant correlation between the average fraction correct by target observed in the human data, and average fraction correct by target simulated by the model (Figure 1E, Spearman’s *ρ* = .25, *p* = .02). In other words, when the model does find the target, it is more likely than chance to find the same targets that humans do.

#### Alignment in Relative Looking Time Depends on Representation.

**Figure 7.**
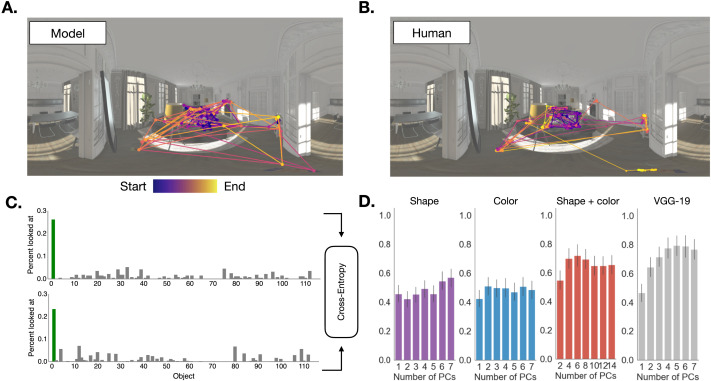
**Comparison between agent and human behavior**. **A:** Gaze trajectories obtained by simulating agent behavior from the *Fixate_MAP* policy 8 times in one of the living room scenes. **B:** Raw gaze trajectories of 8 participants searching in the same scene. **C:** Relative looking times to all objects for the living room scene for agents (top) and humans (bottom). Agents were compared based on the cross-entropy match between simulated and empirical relative looking time distributions. The green bar denotes percent looked at the target object. **D:** Cross-entropy relative to baseline between simulated and empirical relative looking time distributions, as a function of state representation and number of principal components. Higher bars indicate better fit. Note that for the “Shape and color” representation, the effective number of principal components used was twice each value shown on the horizontal axis for the “Shape” and “Color” representations. For example, the agent that simulated behavior corresponding to the first red bar had access to one shape and one color feature (two in total).

The *Fixate_MAP* policy can be treated as a fully generative model of human gaze in naturalistic settings. To ask whether human gaze patterns correspond to *Fixate_MAP*, we simulated gaze trajectories from the policy, and compared them to human gaze data (Figure 7). For instance, in the scene shown in Figure 7, both the model (Figure 7A) and humans (Figure 7B) tended to first look at the couch and coffee table, then at the object on the mantelpiece, then at the objects on the floor, until finding the target on the side table.

As a quantitative model comparison, we computed the cross-entropy between the simulated and observed relatively looking time distributions across all objects in a scene (Figure 7C, Comparing Human and Agent Policies section, higher cross-entropy indicates better model fit). To test for cross-entropy differences between human and model as a function of representation, we fitted a linear mixed effects model that included fixed effects for feature space (shape, color, shape and color and VGG-19) and number of principal components, and random intercepts for participant. The outcome variable was the cross-entropy between: (1) the simulated relative looking time distribution generated by running the model forward on the experiment; (2) the empirical relative looking time distributions obtained from the human participants. By varying the representation the model acts on, we can investigate the contribution of different features to driving human visual search.

An omnibus Type III test revealed a main effect of feature space, such that feature space significantly predicted differences in cross-entropy, (*χ*^2^(3) = 132.91, *p* < .0001). Including number of principal components as a predictor also improved model fit, (*χ*^2^(6) = 246.46, *p* < .0001), suggesting that the number of principal components used in the belief state impacts cross-entropy. Interestingly, adding the feature space × number of principal components interaction also significantly improved model fit (*χ*^2^(18) = 174.42, *p* < .05), suggesting that how well the model aligns with human behavior depended on how many principal components were used.

When comparing marginal means post-hoc comparisons, we found that cross-entropy between the human and model distributions was significantly greater for VGG-19 than for the Shape and Color feature space (*b* = 0.049, *SE* = 0.00985, *z* = 4.970, *p* < .0001; grey vs. red in Figure 7D). Finally, a targeted contrast comparing the best VGG-19 agent to the best Shape and Color agent (highest grey bar vs. highest red bar in Figure 7D) showed that even accounting for number of PCs used, the VGG-19 feature space was a significantly better predictor of the cross-entropy between model and human looking time distributions (*b* = 0.1, *SE* = 0.026, *z* = 3.84, *p* < .005). In sum, of the representations that we tested, agents that learned over the VGG-19 representation and converged to *Fixate_MAP* best matched humans in terms of which objects they fixated on.

#### Alignment in Gaze Transition Structure Depends on Representation.

**Figure 8.**
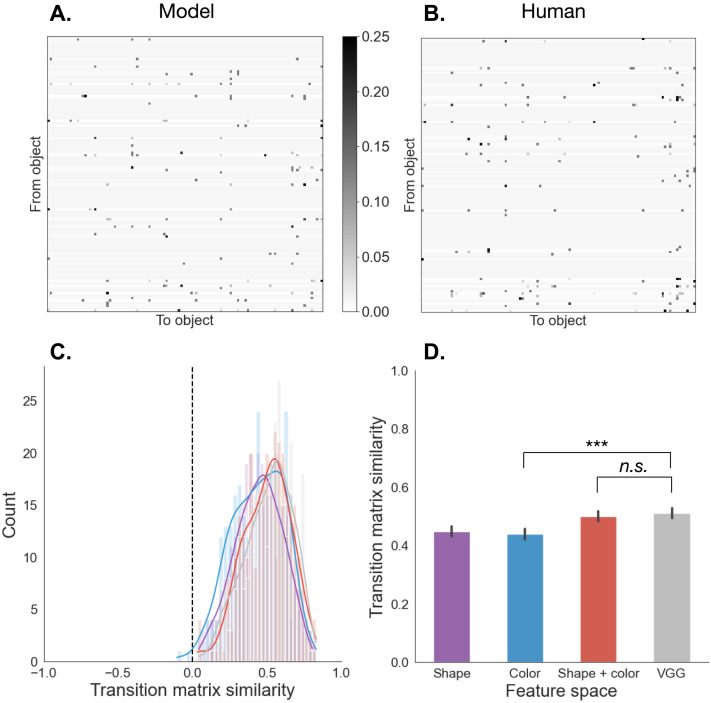
**Analysis of transition structure**. **A:** Simulated transition matrix (averaged across 8 agents) for the scene shown in Figure 7A. **B:** Empirical transition matrix (averaged across 8 participants) for the scene shown in Figure 7B. **C:** Histogram of average transition matrix similarity between model and humans for all scenes. Zero (shown as a dotted line) is equivalent to the transition matrices being no more similar than what would be expected by random chance. **D:** Average transition matrix similarity as a function of feature space.

While useful as a coarse measure of model fit, cross-entropy on its own is agnostic to how well the *Fixate_MAP* policy captures the mechanism for transitions between fixations. To address this, we conducted an additional analysis directly comparing between the transition structure in the human data, and the transition structure generated by the model (Figure 8).

We first computed an empirical transition matrix for each scene, averaged across all participants who searched in that scene. We then generated synthetic data from the best-performing model for each feature space (Figure 7D), and computed a simulated transition matrix for each scene, averaging across the equivalent number of agents. We converted these into distance matrices (based on Euclidean distance), and then performed a Mantel test. In this context, a Mantel test asks whether patterns of transition similarity in one system (humans) are similar to those in another system (agents). The resulting correlation coefficient can be thought of as a measure of the strength of this similarity. Notably, it gives a global test of association, and is not focused on one specific transition, but whether the overall transition structure is similar. This is useful in our setting, in which transitions occur across a large number of objects and are assumed to be generated from a stochastic policy.

Sample transition matrices from agents and humans for one scene are shown in Figure 8A and Figure 8B respectively. We found that for all feature spaces, the average similarity in transition structure between agents and humans is significantly above zero (Figure 8C). This result indicates that *Fixate_MAP* captures human fixation transitions better than expected by chance. A mixed-effects analysis further revealed a main effect of feature space (*χ*^2^(3) = 80.5, *p* < .0001). In other words, as was the case for cross-entropy, the feature space modulated how well the model can match structure in human fixation transitions. Finally, in a post-hoc test, we found that the while the VGG-19 feature space performed significantly better than both Color (*b* = 0.072, *SE* = 0.01, *df* = 897, *t* = 7.22, *p* < .0001) and Shape (*b* = 0.064, *SE* = 0.01, *df* = 897, *t* = 6.41, *p* < .0001) separately, it did not significantly improve upon the conjunction of Shape and Color (*b* = 0.011, *SE* = 0.026, *df* = 897, *t* = 1.08, *p* = 0.70). Taken together, these results suggest that how well *Fixate_MAP* can capture transition structure in fixations also depends upon the choice of representation.

## DISCUSSION

### Summary

In this paper, we present a resource-rational analysis of human visual search in virtual reality environments. We cast eye movements as goal-directed actions for gathering information. We specify a meta-level Markov Decision Process (meta-MDP) that optimally trades off long-term utility (defined by the goal of target retrieval) with the cost of information gathering (how many fixations are needed to reach the goal). To generate candidate state representations for the meta-MDP, we leverage shape and color annotations of virtual objects, as well as a pre-trained deep convolutional neural network (CNN) optimized for image classification. We then train deep reinforcement learning agents to solve the meta-MDP, and compare the learned policy with human gaze trajectories.

Agents learned a belief-guided policy that fixates the object most likely to be the target (*Fixate_MAP*). Even with minimal representational assumptions—spatial contiguity and perceptual similarity across multiple object features—*Fixate_MAP* reproduced several key features of human behavior. A comparison of performance and reaction time between humans and agents revealed evidence that people’s eye movements are consistent with *Fixate_MAP*, and also sensitive to the choice of representation. In particular, the policy best matched human gaze behavior when the state representation was defined by object embeddings encoded in the CNN. Taken together, our results show that naturalistic visual search can be understood as resource-rational sequential decision-making over a rich representation of the visual environment.

### Contributions

Our work shows that optimal models of visual search developed for highly simplified 1D Gaussian IID stimuli (Najemnik & Geisler, 2005) remain near-optimal even in complex, naturalistic settings. Prior decision-theoretic accounts have framed goal-directed eye movements as sequential actions taken to reduce uncertainty about the environment (Acharya et al., 2017; Butko & Movellan, 2008; Hayhoe & Ballard, 2005; Hoppe & Rothkopf, 2019; Najemnik & Geisler, 2005; Sprague & Ballard, 2003). In this framework, visual search is understood as a control policy optimized to detect a target’s location under sensory uncertainty. Our findings extend this view by demonstrating that the same principles apply robustly to rich, real-world scenes.

Our formulation provides a complementary perspective on visual search that differs from previous POMDP accounts (Acharya et al., 2017; Butko & Movellan, 2008; Hoppe & Rothkopf, 2019), which capture how an agent behaves when parts of the external environment are hidden (e.g., due to occlusion). In contrast, a meta-MDP treats uncertainty as arising from an agent’s internal state and models how agents learn and select among mental actions (Callaway et al., 2024; Dayan, 2012). This distinction frames gaze allocation as part of a broader space of internal computations involved in search. Although our results focus on policies defined over objects and object features, the same meta-MDP structure can be naturally extended—for example, to joint actions that select both an object and the subset of features included in the belief state (a form of feature-based attention). Solutions learned in such extensions could serve as hypotheses about the internal structures humans might acquire when solving related tasks, offering a path toward richer models of visual decision-making.

Our results suggest that naturalistic structure plays a central role in shaping search policies. Prior work has shown that in certain environments the optimal policy can deviate substantially from *Fixate_MAP* (Hoppe & Rothkopf, 2019). Such discrepancies may reflect idiosyncrasies of specific experimental paradigms that are comparatively rare in richer, more naturalistic tasks. Many sequential information-sampling models have been evaluated in highly simplified environments whose statistical structure is designed to meet the assumptions of particular algorithms (Najemnik & Geisler, 2005). But humans’ efficiency in real-world search derives, at least in part, from sensitivity to the richer structure present in natural scenes. Our findings are consistent with this view, showing that human-like search policies emerge from resource-rationality assumptions when such structure is incorporated into the model.

Finally, our formulation integrates rich CNN-based visual representations into a sequential decision-making framework, enabling a model of naturalistic visual search that neither approach supports on its own. CNNs provide plausible models of human visual processing (Cohen et al., 2020; Kriegeskorte, 2015; Yamins et al., 2014) and capture high-level structure predictive of human judgments (Fan et al., 2018; Kubilius et al., 2016; Lake et al., 2015). They also underpin many saliency models (Jia & Bruce, 2020; Kümmerer et al., 2016; Linardos et al., 2021), but typically predict aggregated fixation distributions rather than temporally structured scanpaths (Jiang et al., 2016; Kümmerer et al., 2018). Embedding CNN-derived scene structure within a meta-MDP unifies these perspectives, preserving representational richness while providing a principled account of the sequential, goal-directed dynamics of human search.

### Future Directions

Our framework opens the door to systematically identifying which visual features guide search in naturalistic environments. Because the meta-MDP allows ablations of the model’s state representation, future work can directly test which features—color, shape, texture, or higher-order combinations learned by ANNs—are necessary to reproduce human search strategies. This provides a principled way to evaluate feature-guidance theories, in which task goals activate a relevant feature space while search proceeds serially over objects (Treisman & Gelade, 1980; Wolfe & Horowitz, 2017). More broadly, this approach offers a path toward mapping the features that humans rely on when searching in complex visual scenes.

A limitation of the current model is that its state representation does not incorporate higher-order information such as scene guidance or semantic knowledge (Henderson, 2020; Võ, 2021). This mismatch between our representational assumptions and those involved in fully naturalistic search may help explain the differences we observed between human and model performance when the target object and room context were specified. Humans routinely rely on inductive biases in real-world scenes—for example, using “anchor objects” to infer likely locations of other items (Boettcher et al., 2018), or prioritizing surfaces that are semantically appropriate for the target (e.g., looking for a toaster on a counter rather than a fridge). Such biases may have influenced behavior in our experiment even though objects were placed randomly, as assumed by the current model. Future extensions of the meta-MDP could capture these effects by incorporating scene semantics or object co-occurrence statistics into the state representation (Yang et al., 2020).

Finally, our model assumes that gaze is allocated to balance reward and uncertainty while minimizing fixation cost (Hayhoe & Ballard, 2014). Yet humans are unlikely to sample all features uniformly. Depending on the task goal, certain features may be more informative for target detection than others (Ho et al., 2022; Leong et al., 2017; Niv, 2019; Zelinsky et al., 2020). For example, color may be helpful when searching for clothing but less relevant when locating car keys. How such feature hierarchies emerge, and how they shape gaze allocation, remains an open question (Kurenkov et al., 2021). The framework introduced here could be extended to explore these processes, either by expanding the action space to allow sampling at both the object and feature levels or by allowing the cost of sampling different features to vary with the task.

In this paper, we take a first step toward a computational account of human goal-directed action selection in fully immersive virtual environments. We show that naturalistic visual search can be understood through the lens of resource-rational analysis, providing a reinforcement learning framework for discovering policies that maximize search efficiency while minimizing information-gathering costs. Instantiating this policy in a representational space that captures key aspects of naturalistic visual scenes, we find that human gaze patterns align closely with the model’s predictions. Together, these results demonstrate that resource-rational analysis can serve as a predictive framework for behavior in ecologically valid settings.

## METHODS

### Immersive Visual Search Task

Classic studies of visual search are designed to test the human ability to use specific features to find objects (Wolfe, 2010). While these studies have identified several features of the environment that drive eye movements, such stimuli do not reflect the multidimensional structure of real-world environments. To address this, we conducted a study of visual search in virtual reality (VR). 26 participants viewed VR scenes generated with the Unity game engine through a head-mounted display (HTC Vive VR), equipped with a Tobii Pro VR eye tracker (sampling frequency: 120 Hz), while holding a handheld controller.

Data collection took place between May 2018 and November 2019. Participants provided informed consent consistent with the Declaration of Helsinki. Experimental sessions lasted approximately 60 minutes, for which we compensated participants $50. We excluded data from 5 participants for data quality issues (3 due to incomplete data; 2 due to calibration issues resulting in timing discrepancy between visual stimulus and gaze data), yielding a dataset of 21 participants.

Each participant performed 300 trials of visual search for a target in a cluttered room with 55–112 distractors and under an 8 second deadline. We selected 6 possible room contexts as stimuli, from the pre-designed ArchVizPRO Interior Vol.3 template, commercially available in the Unity Asset Store. This asset realistically renders an apartment in an immersive setting. We determined 5 possible viewpoints in each room by manually manipulating the position of the in-game camera in Unity. We specified 10 target / distractors sets from a set of pre-designed 113 objects, each commercially available in the Unity Asset Store as single assets (e.g., “apple”) or within asset bundles (e.g., “Fruit Pack”).

A trial was defined by a combination of one of 6 possible rooms (kitchen, living room, bathroom, study or one of two bedrooms), one of 5 pre-determined viewpoints, and one of 10 pre-generated target/distractor sets. Each participant experienced the set of 300 unique trials this procedure generated in a different random order. On some trials participants were presented with visual recommendations represented as transparent blue heatmaps overlaid on the visual display. These heat maps changed in real time and were designed to aid participants in their search by recommending likely areas of the scene where the target could be found. Each participant received assistance from these visual cues across 100 random trials during the study. Because this modification was exploratory, the data gathered from the assisted trials are not included here.

Each trial began by showing the target object for 4 seconds in an empty, white scene. The participant was then immersed into the room from a perspective selected *a priori*. At any time during the 8-second search period, the participant could report having found the target by pointing and clicking with a handheld controller. If the participant did not find the target with the 8-second period, the trial ended.

We determined the identity, size, location and orientation of the target object and distractor objects with a number of constraints: (1) each object rested on a stable surface (e.g., floor, table, etc); (2) none of the distractors had the same object identity as the target, or were too similar; (3) at least 50% of the target was visible from the participant’s viewpoint; (4) the visible area of the target was at least 3 degrees of visual angle squared; (5) the size of each object was determined by uniformly scaling each Unity prefab (i.e., object template) obtained from the Unity Asset store by a random amount between 1× and 2× its original size. This ensured that the original proportions were preserved, but each object was rendered as uniformly bigger or smaller. The overall effect of this object generation procedure was that location, orientation, absolute and relative object size were randomly determined.

### Feature Extraction

#### Explicit Method.

Our explicit feature extraction approach draws from work in visual cognition showing that eye movements are guided by objects and object shape, color and texture (Wolfe & Horowitz, 2017). We quantified shape as the D2 distribution over the 3D mesh of each object (Osada et al., 2002; Figure 2A, left). And we quantified color by converting the 2D texture of each object to the CIE L*a*b* color space and extracting the A (green-red) and B (blue-green) channels (Figure 2A, right).

The D2 shape distribution and CIE L*a*b* color representation both yield high-dimensional representations of the shape and color of each object, constrained by the number of times pairs of points are sampled from the mesh (in the case of D2) and the pixel size of the texture (in the case of CIE L*a*b*).

To compute the shape distribution, we randomly sampled 500,000 pairs of points on the mesh surface and computed the Euclidean distance between each pair. We further reduced this distribution to a histogram with 100 bins, applying PCA to a shape matrix consisting of *N*_objects_ × *N*_bins_. For converting color texture to CIE L*a*b*, we resized each texture image to 500 × 500 pixels, and concatenated the A and B channels. To preserve spatial color information, we opted not to histogram this representation, applying PCA directly to a color matrix of dimensionality *N*_objects_ × *N*_pixels_.

Due to its ease of computation and robustness in the presence of rotations, translations and other shape perturbations, the D2 distribution has been widely used in the computer graphics and computer vision literature as a metric for discriminating between shapes of different classes (Osada et al., 2002). Importantly, data from human similarity judgments suggests the D2 distribution captures some aspects of human shape perception (Clark et al., 2006).

The CIE L*a*b* color space describes all the colors visible to the human eye, and can thus serve as a metric space for color similarity that roughly matches that of human color perception. Because color was extracted from 2D texture files, this representation is likely to also include some textural elements such as smoothness or graininess.

#### Implicit Method.

Recent work has shown that CNNs capture some aspects of the human visual architecture and processing (Yamins et al., 2014). 2D images of objects can be represented as points in multidimensional feature spaces learned by CNNs (known as “embeddings”). These embeddings can then be used downstream to support different tasks such as categorization, typicality judgments or visual production (Battleday et al., 2020; Lake et al., 2015; Long et al., 2019). We used a standard pre-trained Keras implementation of the VGG-19 architecture (Simonyan & Zisserman, 2014) to extract embeddings across different depths of the network (Figure 2B).

Specifically, we passed a 224 × 224 pixel image of each object viewed from a canonical viewpoint through a Keras implementation of the pre-trained VGG-19 architecture. Running the model forward, we obtained a multidimensional embedding of all objects in the experiment. How predictive this embedding was of gaze depended on which layer of the network we used. VGG-19 consists of two modules: a perceptual module that alternates blocks of convolutions with pooling layers; and a classification module consisting of two fully connected layers and a softmax layer. We analyzed feature activations in the second-to-last fully-connected layer (FC-4096, dimensionality 1 × 4096), as well as the last pooling layer (Max-Pool 5, dimensionality 7 × 7 × 512). For the latter, we averaged and flattened the 512 spatial maps, yielding an embedding with 49 dimensions for each object.

#### PCA.

To further reduce the dimensionality of our feature space, we separately applied PCA to each feature type and computed low-dimensional projections in the space defined by the principal components. For the shape and color features, PCA was applied over matrices in which rows indexed objects, and columns indexed bins of either the shape histogram or raw pixel-level color information associated with each object. For VGG-19 features, columns indexed features of each object’s embedding obtained from the Max-Pool 5 layer. Across all features, we computed the principal components needed to explain 95% of the variance in feature space.

### Full Meta-MDP Specification

A meta-MDP consists of a latent state, beliefs over the latent state, actions, a transition function and a reward function. The full meta-MDP for visual search can be specified as follows:

- **Latent state**: The latent (unknown) state is represented as a matrix *A* of dimensionality *N*_*o*_ × *N*_*f*_ such that the value of feature *f* for object *o* is *A*_*of*_, where *N*_*o*_ is the number of objects and *N*_*f*_ is the number of features in a particular representational space. In line with previous work (Diuk et al., 2008; Dubey et al., 2018), the agent has knowledge about the *N*_*o*_ objects present in each scene, but not about the features of those objects. The matrix *A* is assumed to be a fixed property of the environment.
- **Beliefs**: The agent represents the environment as beliefs over features in *A*. For tractability, we assume independent Gaussian beliefs: beliefs are represented with mean and precision matrices of dimensionality *N*_*o*_ × *N*_*f*_, *F* and *J*, such that:
  $$p\left(A_{\text{of}}\right)=𝒩\left(F_{\text{of}},1/J_{\text{of}}\right)$$ (1)
  Here, *F*_*of*_ represents the mean belief for a particular feature *f* of object *o*, and *J*_*of*_ represents the precision of that belief (so that the variance is 1/*J*_*of*_). At the beginning of each episode, we initialize the beliefs with a broad, uninformative prior:
  $$\begin{array}{l} F_{\text{of}}∼𝒩\left(0,0.01^{2}\right),\\ J_{\text{of}}^{\left(0\right)}=0.01. \end{array}$$
  This initialization sets all feature means close to zero with small jitter, and assigns low precision (*J*_*of*_ = 0.01), which corresponds to high belief uncertainty (variance 1/*J*_*of*_ = 100). Thus, the agent starts with a highly uncertain (effectively uninformative) prior over feature values.
- **Computations**: A computation corresponds to looking at the center of object *o*.
- **Actions**: An action corresponds to performing a computation.
- **Transition**: Formally, a computation takes a measurement *X* of the features of objects near the center of gaze, and incorporates that measurement into the belief using Bayesian cue combination. To specify this transition:
  1. Compute an object attentional mask *g*_*o*_, in which the attention paid to all objects in the scene exponentially decreases with their distance to *o*. This mask can be thought of as a “fovea.” To implement it, we used a von Mises–Fisher kernel that scores how aligned each object location in 3D space *x*_*o*_ is to the location of the current fixation *μ*:
    $$g_{o}={{\text{scale}}_{go}}^{*}\exp\left[\left(x_{o}^{T}\mu -1\right)\cdot k\right]$$ (2)
    Here, scale_*go*_ and *k* are free parameters that govern the properties of the kernel: scale_*go*_ adjusts the overall strength of the output, and *k* controls how sharply the output responds to differences in location.
  2. Compute the measurement precision *J*_*meas*_ = *g*_*o*_1^*T*^ such that the features of objects near the center of gaze have the highest precision. If an object is currently being fixated, alignment is maximal and information about the object is sampled with full precision. Misalignment between object and fixation location leads to exponential decay in sampling precision.
  3. Independently sample a measurement from the true latent state:
    $$X∼𝒩\left(A,1/J_{\text{meas}}\right)$$ (3)
  4. Perform the Bayesian update
    $$F\leftarrow \frac{F⊙J+X⊙J_{\text{meas}}}{J+J_{\text{meas}}}$$ (4)
    and *J* ← *J* + *J*_*meas*_, where ⊙ represents the element-wise product.
  5. **Reward**: The agent incurs a cost −*c* for each computation, where *c* is a free parameter governing how much the agent gets penalized for each additional fixation. When the agent terminates computation, it calculates a posterior over which object *o* is the target given its beliefs,
    $$p_{o}\propto \exp\left(\frac{1}{2}\sum_{f}\left[\log\left(1+J_{\text{of}}\right)+\frac{J_{\text{of}}}{1+J_{\text{of}}}F_{\text{of}}^{2}-J_{\text{of}}{\left(F_{\text{of}}-f_{f}^{\text{target}}\right)}^{2}\right]\right)$$ (5)
    This calculation assumes that the true values of object features are all mutually independent and Gaussian with mean 0 and variance 1. The agent then selects *argmax*[*p*_*o*_] as its target report and receives a final reward of *R* = 1 if this is correct and *R* = 0 otherwise.
  6. **Termination**: We denote ⊥ as a special computation for which search terminates whenever *max*[*p*_*o*_] exceeds a threshold *θ*. Here, *θ* is a free parameter that governs how confident the agent needs to be before terminating search. A summary of model parameters and how they were set for each experiment is shown in Table 1.

**Table 1.** Parameters of the meta-MDP.

**Parameter**	**Value**	**Expt.** Learning a Resource-Rational Search Policy	**Expt.** Effects of Representation on Agent Performance	**Expt.** Predicting Human Behavior
Initial mean belief (*F*_*of*_)	𝒩(0, 0.01^2^)	Fixed	Fixed	Fixed
Initial belief precision ($J_{\text{of}}^{\left(0\right)}$)	0.01	Fixed	Fixed	Fixed
Mask scale (scale_*go*_)	3	Fixed	Fixed	Fixed
Mask sharpness (*k*)	200	Fixed	Fixed	Fixed
Cost of computation (*c*)	0.01	Fixed	Fixed	Fixed
Termination threshold (*θ*)	*θ*	Optimized (see Threshold Optimization section)	Optimized	0.998
Lapse rate (*ϵ*)	*ϵ*	–	–	Fit (see Comparing Human and Agent Policies section)

*Note*. The initial mean and precision values provide an uninformative prior over object features. The attentional mask scale and sharpness, as well as the cost of computation, were set to fixed values. These initialization values are considered hyperparameters and are not tuned during training of the deep-RL agent. Model robustness analyses showing effects of hyperparameters on the policy at convergence are shown in Supplementary Information Section 5.8.

### Solving the Meta-MDP

In order to identify the optimal policy for the meta-MDP, we trained artificial neural network agents to maximize the expected cumulative rewards in an ensemble of visual search environments. For each of the 300 scenes presented in the human task, we generated an artificial environment in which the target was the same as in the human experiment. We also created additional environments by considering the same scene, but with a different object assigned to be the target. We only generated such scenes if that object was unique among the object set, resulting in an ensemble of 7022 environments.

Artificial environments were generated from the first six principal components of the objects’ feature representations. To equalize environment size, we added phantom objects to any environment with fewer than the maximum (113), assigning them feature values of zero.

We applied one further modification to the state and action spaces: before passing the belief state to the preprocessing layer, we reordered objects by their posterior rank and applied the same remapping to the action space. Thus, the first rows of the belief state and the first action always correspond to the object currently most likely to be the target.

We implemented and trained the artificial agents in tensorflow (Abadi et al., 2015) with the tf-agents library (Guadarrama et al., 2018). We used proximal policy optimization (PPO) (Schulman et al., 2017) to define the loss function, and trained using Adam (Kingma & Ba, 2014) with exponential learning rate decay. Since PPO is an actor-critic method, we specified two neural networks which only differed in the output layer (Figure 2D). Both networks consist of a preprocessing layer which represents inputs (see below), then a series of 3 densely connected layers of 113 units each, followed by an output layer (a single value for the critic, an action distribution layer for the actor).

The preprocessing layer represents a belief state in the meta-MDP as a list of 4 tensors:

- *F*, a 113 × 6 matrix of the mean feature values.
- *J*, a 113 × 6 matrix with the corresponding precisions.
- *f*_target_, a 1 × 6 vector with the true values of the target object’s features.
- *x*_*o*_, a 113 × 2 matrix with the horizontal and vertical coordinates of each object, normalized so that the screen maps to the interval [−1, 1]. For phantom objects, we set *x*_*o*_ to (0, 0), the screen center.
- *p*_*o*_, a 113 × 1 vector with the output of Equation 5. We set *p*_*o*_ to 0 for phantom objects, then re-normalize to ensure the sum of *p*_*o*_ over non-phantom objects equals 1. Although *p*_*o*_ is a deterministic function of *F*, *J*, and *f*_target_ and therefore provides no additional information to the agent, we include it in the state representation to accelerate learning.

The preprocessor then passes each of these tensors through a single dense layer with 113 units and concatenates the outputs into a single tensor which represents the belief state.

### Ablation Study

To determine how sensitive reinforcement learning agents are to the choice of representation, we performed an ablation study in which we simulated behavior from the *Fixate_MAP* policy over different state representations, using a threshold optimized for the specific state representation (Threshold Optimization section, Supplementary Information Section 5.5). Specifically, we simulated behavior from the agent over shape features only, color features only, shape and color features and VGG-19 features, varying the number of principal components that the agent had access to. And we computed the across-scene average return and number of fixations that *Fixate_MAP* agent obtained given these different state representations.

### Threshold Optimization

The *Fixate_MAP* policy terminates search when any item is deemed sufficiently likely to be the target. Formally, the policy executes the ⊥ action when max[*p*_*o*_] > *θ*, where the threshold *θ* is a free parameter. We set this parameter to maximize the expected total reward attained per trial for each combination of feature space and number of principal components (Ablation Study section). Concretely we defined an objective function that approximates the expected reward as the average reward in 10,000 simulated trials, and optimized this function with a grid search over the range (0.5, 0.999) in logit space (i.e., smaller distances between values near 1). Results are shown in Supplementary Information Section 5.5.

### Statistical Analysis

Mixed-effects models were fit with lme4 (Bates et al., 2015).

### Comparing Human and Agent Policies

To analyze gaze trajectories, we first transformed the raw gaze sample coordinates to the pixel space of 360 degree equirectangular projections from the pre-specified viewpoints in each scene (Sitzmann et al., 2018; Figure 1B). This transformation is necessary in order to simultaneously take into account participants’ head and eye movements. We then annotated each raw gaze sample with the label of the object at the center of gaze. We extracted this object label by recording the object mesh collider hit by a ray in the Unity environment in the direction of gaze. We found that about 50% of gaze samples landed either on task-relevant objects (i.e., the target or a distractor), suggesting that objects are a strong cue for gaze, consistent with previous findings (Einhäuser et al., 2008). In this paper, we restrict analysis to those gaze samples which hit task-relevant objects.

Because the model outputs fixations in object space, we did not segment trajectories into fixations and saccades. This decision sidesteps challenges associated with saccade detection algorithms that are developed for head-fixed participants viewing 2D screens, and may not readily extend to free viewing in 3D virtual reality environments (cf. Diaz et al., 2013). To compare the policies of the trained agent with those of human participants, we simulated 20 fixation trajectories per scene. This simulation assumed a fixed cost parameter and threshold.

We then computed the proportion of time (in units of raw fixations) that the agent looked at each object for a given scene. To compare each resulting scene-wise probability distribution with the empirical distribution, we used cross-entropy, fitting a lapse rate *ϵ* for each individual participant (Supplementary Information Section 5.7). We report this cross-entropy metric averaged across scenes and participants, and subtracted from a baseline model which assumes uniform relative looking times across all objects (i.e., maximal cross-entropy). The higher this difference, the more closely the distribution of objects fixated by the model matches the distribution of objects fixated by participants.

To obtain a reaction time prediction from the model, we make some simplifying assumptions about how the model’s “fixations” translate to human fixations. Specifically, we assume a linear relationship between number of fixations and the RT on a given trial. To generate reaction time (RT) on each trial, we “fit” the model to the mean RT from the experiment. That is, we first compute the empirical mean RT *m* (4.55 s). We next run the model forward with the best fitting parameter settings, and obtain the average number of fixations the model produces *f* (12.39). Finally, we compute a time constant *τ* as the ratio between the empirical mean RT and the simulated mean number of fixations, *τ* = *m*/*f*. The model then simulates the reaction time on each trial by multiplying *τ* by the number of fixations on that trial.

## ACKNOWLEDGMENTS

We are grateful to Ruta Desai, Kara Emery, and Todd Gureckis for insightful discussions on the work presented in this manuscript.

## FUNDING INFORMATION

This research was conducted during internships at Meta (A.R. and B.v.O.) and subsequently supported by a Facebook/Meta research grant awarded to A.R.

## AUTHOR CONTRIBUTIONS

A.R.: Conceptualization; Data curation; Formal analysis; Funding acquisition; Investigation; Methodology; Software; Visualization; Writing – original draft. B.v.O.: Conceptualization; Data curation; Formal analysis; Investigation; Methodology; Software; Writing – original draft. F.C.: Formal analysis; Methodology. T.L.G.: Conceptualization; Funding acquisition; Supervision; Writing – review & editing. J.M.H.: Conceptualization; Funding acquisition; Supervision; Writing – review & editing.

## DATA AND CODE AVAILABILITY STATEMENTS

Analysis code is available at https://github.com/angelaradulescu/modeling-eye-movements-vr and data are available upon request.

## Supplementary Material


