# Supplementary material for: A Resource-Rational Account of Human Eye Movements During Immersive Visual Search
Source: Open Mind (Camb). 2026 Feb 1;10:91–117. doi: 10.1162/OPMI.a.322 (PMC13053022; doi:10.1162/OPMI.a.322)
Supplement: Supplementary file 1 [file opmi-10-91-s001.pdf]

5 SUPPLEMENTARY INFORMATION

5.1 *Layer specificity analysis*

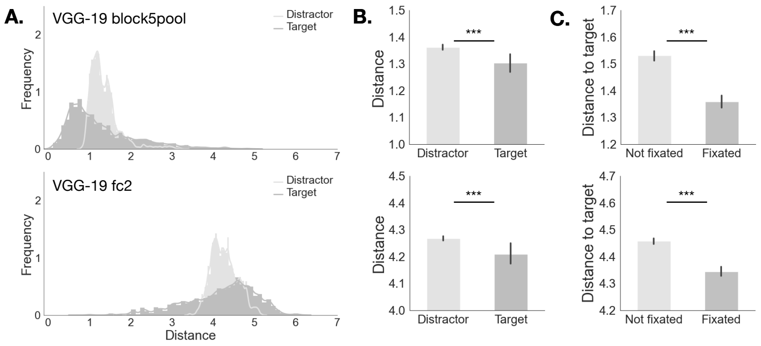

Figure S1. **A**: Distribution of distances between the features of currently fixated object and the features of either the target (darker shade) or the average features of all distractors (lighter shade). Shown here are the distributions computed from the VGG-19 embedding, using either the last perceptual layer of the network (top) or the second-to-last classification layer (bottom). **B**: Means and 95% CIs of the distributions in A. **C**: Distance to target for all objects in a scene as a function of whether the object was fixated or not. Distance was computed pairwise between object as $\frac{(1-\rho)}{2}$, where ρ is the Spearman correlation coefficient. ***: $p < .0001$.

To test which layer is a better predictor of gaze as a function of target similarity, we ran two mixed-effects models: one which included "is target" vs. "is-not-target" as a predictor; and a second which included "fixated" vs. "is-not-fixated" as a predictor. In both models, we included an interaction with layer, and random intercepts for each participant. The first model tests the hypothesis that distance to the fixated object differs depending on whether it is computed relative to the target, or relative to the average of all distractors. The second model tests the hypothesis that the distance to target differs between objects that were fixated and those that were not. A significant interaction would indicate that the layer from which the embedding is computed significantly changes the magnitude of the difference.

Adding the layer x target interaction significantly improved fit for the first model ($\chi^2(1) = 8.67, p < 0.005$). A post hoc test showed that, as expected, distance to fixated object was significantly higher for non-targets than for targets ($b = 0.07, SE = 0.001, z = 64.45, p < .0001$). This distance was higher for the classification layer ($b = 2.9, SE = 0.001, z = 2589.5, p < .0001$). In other words, when using the classification embedding, fixated objects were more similar to the target than to the average distractor. Adding the layer x target interaction also significantly improved the fit of the second model ($\chi^2(1) = 75.74, p < 0.0001$). A post hoc test revealed that, as expected, distance to target was higher for non-fixated than for fixated objects ($b = 0.14, SE = 0.003, z = 42.20, p = p < .0001$). Distance to target between fixated and non-fixated objects was higher for the classification layer ($b = 2.96, SE = 0.003, z = 868.4, p < .0001$). In other words, when using the perceptual embedding, fixated objects were even more similar to the target than non-fixated objects. Since the perceptual embedding was a better predictor of which objects were fixated as a function of target similarity, we used this embedding in subsequent analyses.

5.2 PCA validation

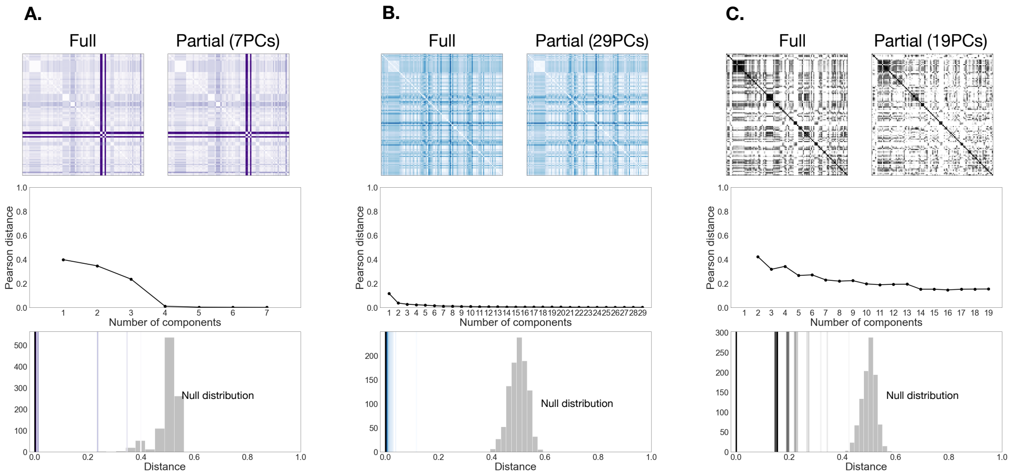

Figure S2. To validate the choice of PCA as a dimensionality reduction step in our model, we generated representational dissimilarity matrices (RDMs) for all the objects in used in the experiment Kriegeskorte et al. (2008); Venkatesh et al. (2020). For each object we computed the pairwise Euclidean distance between shape and color vectors, and the pairwise Spearman distance between VGG-19 embeddings. We used used either the full representation (RDM-full), or a partial representation based on a restricted number of principal components (RDM1, RDM2, etc). An example comparison between the full RDM and a partial RDM is shown in the top row for shape (A), color (B) and the VGG-19 embedding (C) respectively. We found that for all representational spaces, the distance between full and partial RDMs decreases as the number of components used increases, middle row). We then performed a permutation test to obtain a null distribution for the Pearson distance between the intact and randomly permuted full RDM. This distribution provides an estimate of the upper bound on the Pearson distance we might expect between different RDMs. Taking the distance of the full RDM to itself as a lower bound, we computed and plotted the distance between RDM-full, RDM1, RDM2, etc. We found that similarity is largely preserved for low-dimensional projections in the space of principal components. For shape, color and the VGG-19 embedding, all RDMs were well outside the null distribution obtained via permutation testing (bottom row). In other words, PCA preserves the representational structure of each candidate feature space.

5.3 Verifying normality assumptions

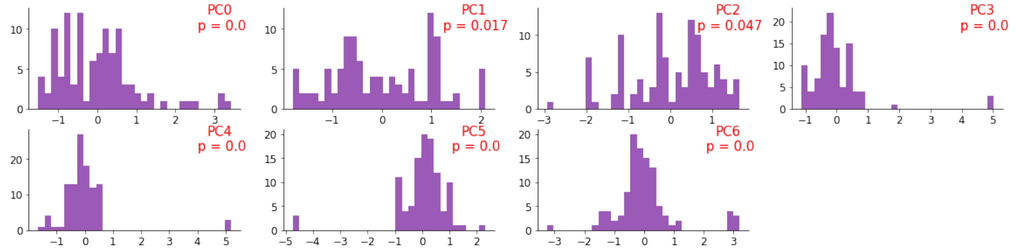

Figure S3. Histogram of shape features, annotated with p-values for D'Agostino and Pearson's normality test. Red denotes features for which this test was statistically significant (that is, we can reject the null hypothesis that features were normally distributed).

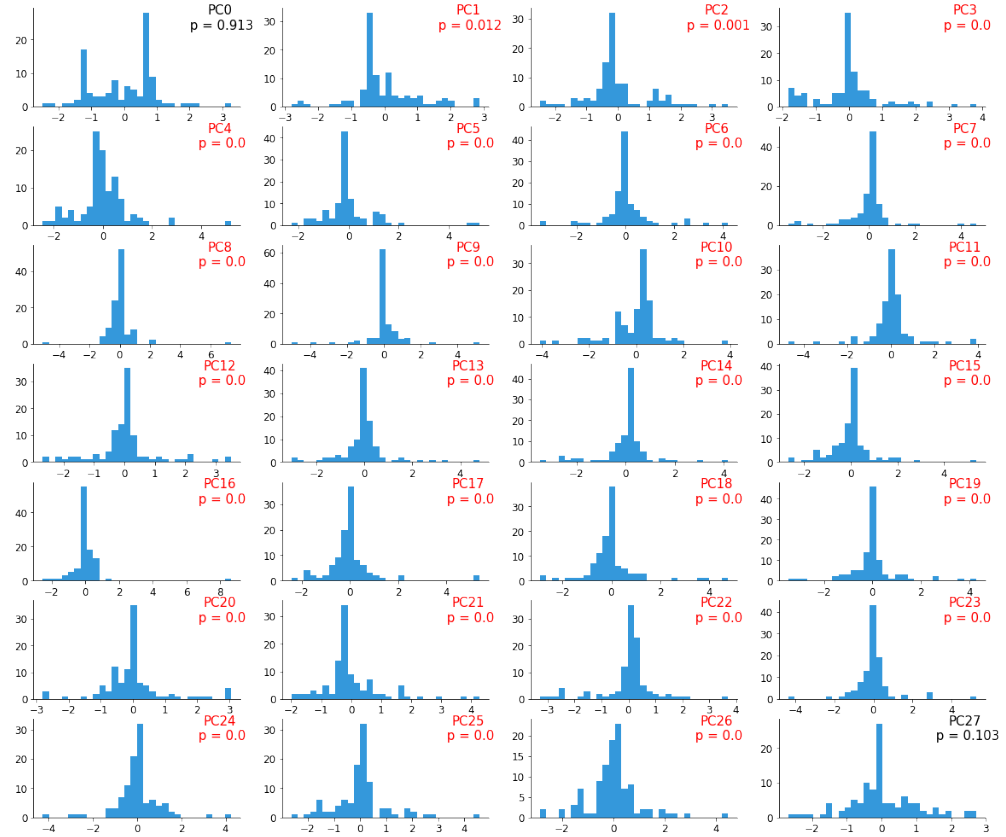

Figure S4. Histogram of color features, annotated with p-values for D'Agostino and Pearson's normality test. Red denotes features for which this test was statistically significant (that is, we can reject the null hypothesis that features were normally distributed).

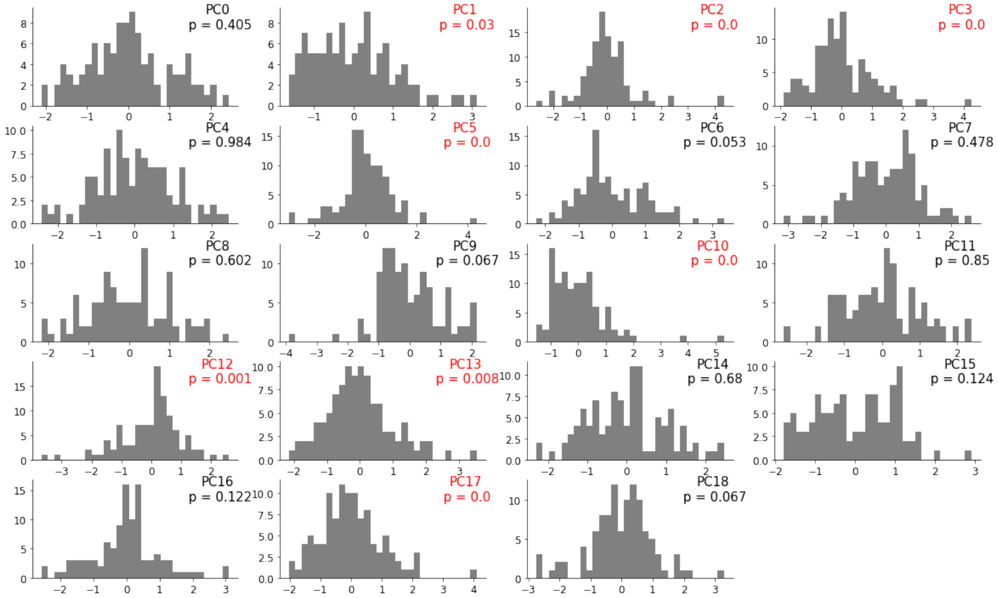

Figure S5. Histogram of VGG-19 features, annotated with p-values for D'Agostino and Pearson's normality test. Red denotes features for which this test was statistically significant (that is, we can reject the null hypothesis that features were normally distributed).

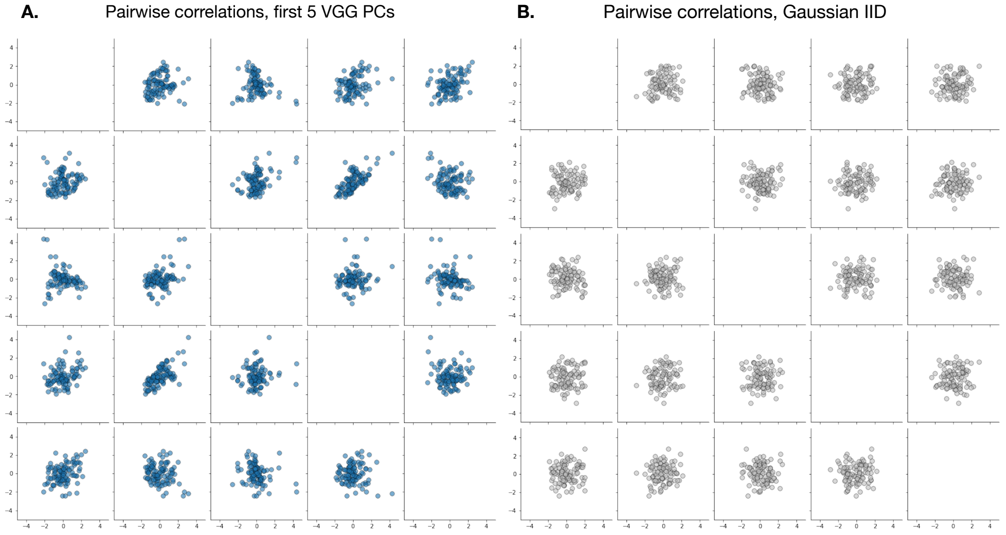

Figure S6. Comparison of pairwise correlations between VGG-19 features (**A**) and randomly generated Gaussian IID features (**B**). If features were Gaussian IID, then we would expect the covariance of any two features to be close to 0.

5.4 Model training results for shape and color feature space.

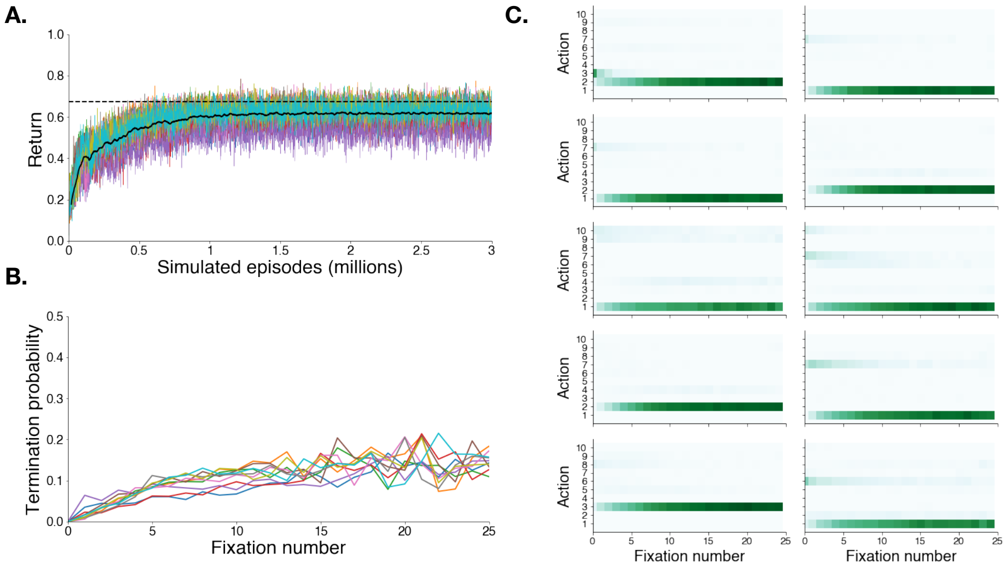

Figure S7. **A**: Learning curves for 10 agents trained to solve the meta-MDP using deep reinforcement learning over the shape and color feature space. As for the VGG-19 representation, all agents converge after about 1 million episodes. The dotted line represents the average return obtained by the *Fixate_MAP* policy (see Results section). **B**: Probability of terminating search as a function of the number of fixations previously taken, for the 10 different networks. **C**: Histogram of chosen action as a function of fixation number for each of the 10 agents. Actions are ordered by the posterior probability that the fixated object is the target. So Action 1 corresponds to fixating on the object most likely to be the target.

5.5 Threshold optimization

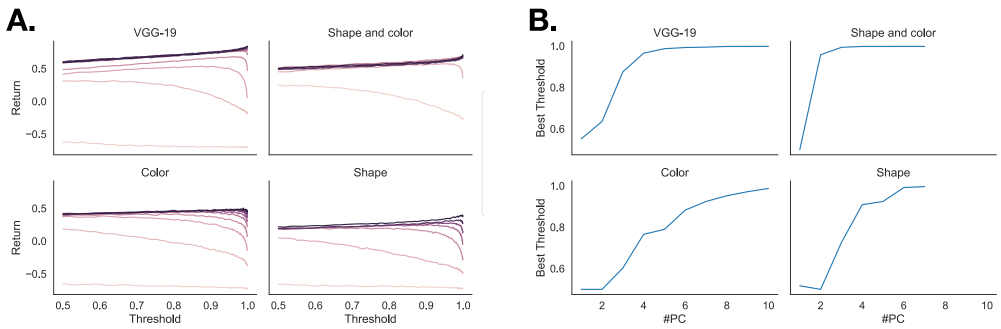

Figure S8. Threshold optimization. **A**: the return attained by the *Fixate_MAP* policy with different feature spaces and threshold values. **B**: the best-performing threshold for each feature space.

5.6 Room context dependence

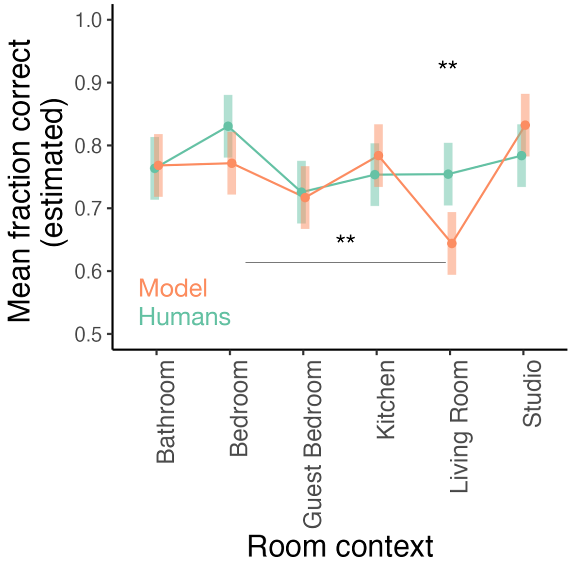

Figure S9. Search performance is context dependent. If search is easier for the model in the same contexts in which it is for humans, we might expect a similar ranking in terms of accuracy across rooms. We fitted a linear mixed effects model to examine the relationship between fraction correct and type of data (real vs. simulated) and room context, including random intercepts for participant (where some "participants" are real participants and some are simulated agents), including an interaction term between data type and context. The plot depicts estimated mean fraction correct as a function of context, compared between model and humans.

We analyzed accuracy using a mixed-effects model with data type (human vs. model), room context, and their interaction as fixed effects. There was no main effect of data type, $\chi^2(1) = 0.02, p = .90$, indicating that humans and the model performed at similar overall levels ($b = 0.005$, $SE = 0.036$, $t(194) = 0.13$). In contrast, we observed a significant main effect of room context, $\chi^2(5) = 12.73, p = .026$, indicating substantial variation in difficulty across contexts. Critically, there was a significant data type $\times$ room interaction, $\chi^2(5) = 17.51, p = .0036$, showing that human–model differences depended on the context. Post hoc comparisons revealed that humans performed significantly worse than the model only in the living room context ($b = -0.110$, $SE = 0.036$, $t(194) = -3.09$, $p = .0023$), with no reliable differences in the other contexts.

To assess whether humans and the model showed similar context-dependent performance, we compared their rank ordering of accuracies across rooms using Kendall's Tau. The association was not significant ($\tau = 0.33, p = .47$), indicating that the models and humans did not prioritize contexts in the same way. One explanation is that human search behavior draws on higher-order semantic priors – such as expectations about which objects are likely to appear in different environments – whereas the model relied solely on object-level perceptual features. Although this representation is suitable for our task, where object placement was random, it would be insufficient in more naturalistic settings where semantic and spatial regularities guide search.

5.7 Individual differences in lapse rate

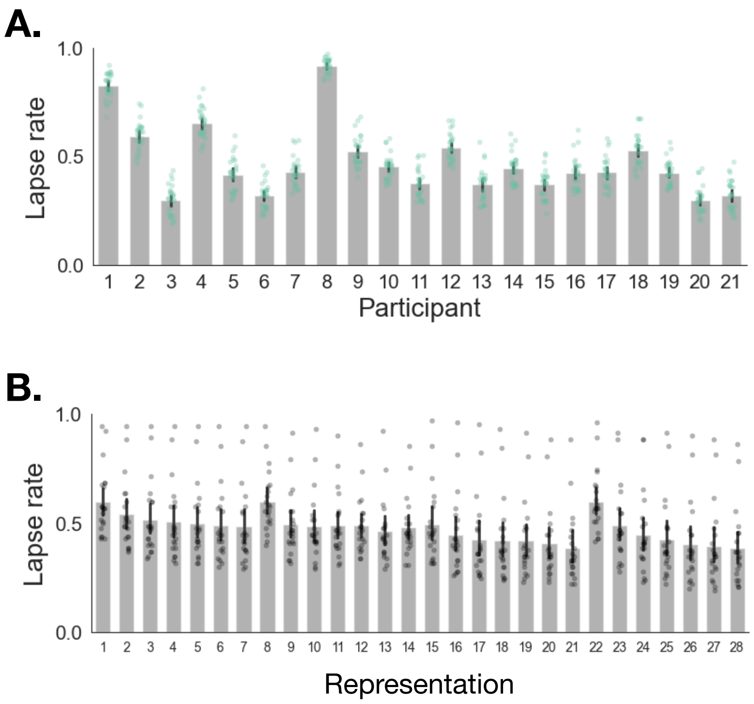

Figure S10. Individual differences in lapse rate. **(A)** To assess individual differences in model fit, we estimated a lapse-rate parameter for each participant, averaged across all the representations (feature space x number of principal components) we tested. Lapse rates showed substantial variability (range: 0.30-0.92), as well as consistency within representations. This indicates that the model captured some participants' behavior more closely than others. Because the lapse parameter reflects both model-unexplained variance and intrinsic stochasticity in gaze choices (analogous to softmax temperature in choice models), higher lapse rates correspond to either noisier behavior or reduced alignment with the model's predictions. **(B)** Average lapse rate across all participants, as a function of representation.

5.8 Model robustness

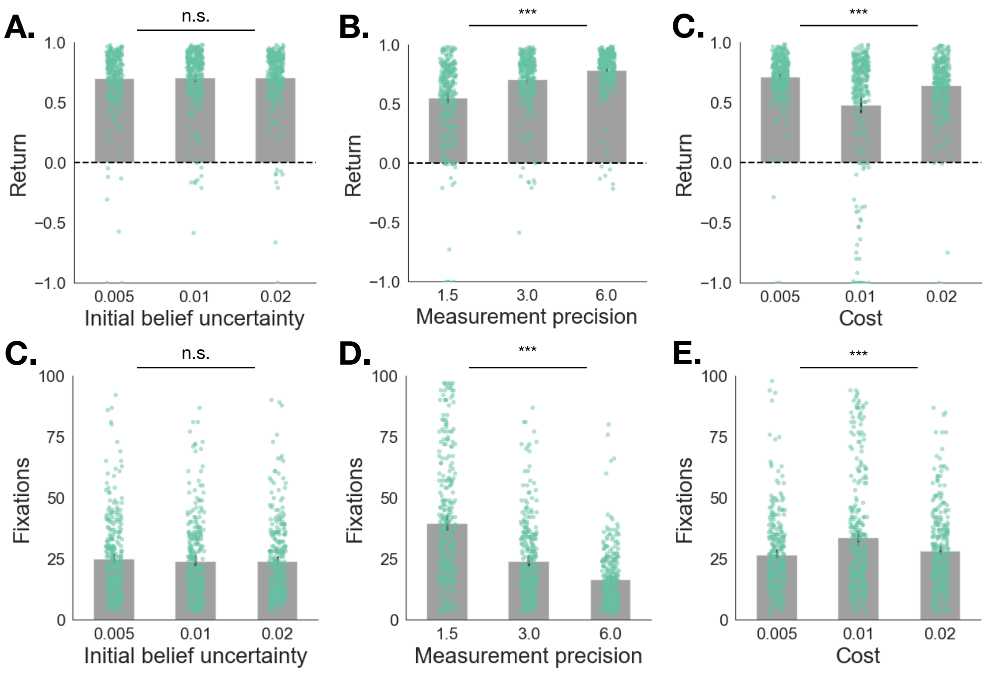

Figure S11. Model robustness. To determine how robust the *Fixate_MAP* policy is to changes in hyperparameters (initial belief precision J_{of}, measurement precision scale$_{go}$ and cost of computation c), we simulated average return and number of fixations, varying each parameter in turn while keeping the others constant at a set baseline: $J_{of} = 0.01$, scale$_{go} = 3$, $c = 0.01$. In all simulations reported here, the shape parameter k, which controls how selectively precision is allocated across objects as a function of feature match, was held fixed; we focused instead on scale$_{go}$, which determines the overall level of measurement precision. **(A)** Average return as a function of initial belief uncertainty. A one-way ANOVA revealed no significant effect of belief uncertainty on return, $F(2, 87) = 0.05,\ p = .95$. **(B)** Average return as a function of measurement precision. A one-way ANOVA revealed a significant effect of measurement precision on return, $F(2, 87) = 60.41,\ p < .0001$. **(C)** Average return as a function of cost. A one-way ANOVA revealed a significant effect of cost on return, $F(2, 87) = 31.10,\ p < .0001$. **(D)** Average fixations as a function of initial belief uncertainty. A one-way ANOVA revealed no significant effect of belief uncertainty on fixations, $F(2, 87) = 0.35,\ p = .70$. **(E)** Average fixations as a function of measurement precision. A one-way ANOVA revealed a significant effect of measurement precision on fixations, $F(2, 87) = 121.69,\ p < .0001$. **(F)** Average fixations as a function of cost. A one-way ANOVA revealed a significant effect of cost on fixations, $F(2, 87) = 11.49,\ p < .0001$. Taken together, these results suggest that the model performance is sensitive to measurement precision and cost, but not to initial belief uncertainty.
